# Mesenchymal Stem Cells for Prophylaxis of Chronic Graft-vs-Host Disease After Haploidentical Hematopoietic Stem Cell Transplant: An Open-Label Randomized Clinical Trial

**DOI:** 10.1001/jamaoncol.2023.5757

**Published:** 2023-12-28

**Authors:** Ruihao Huang, Ting Chen, Sanbin Wang, Jishi Wang, Yi Su, Jing Liu, Yanqi Zhang, Xiangyu Ma, Qin Wen, Peiyan Kong, Cheng Zhang, Lei Gao, Jiang F. Zhong, Li Gao, Xi Zhang

**Affiliations:** 1 Medical Center of Hematology, Xinqiao Hospital, State Key Laboratory of Trauma and Chemical Poisoning, Army Medical University, Chongqing, China; 2 Department of Hematology, 920th Hospital of Joint Logistics Support Force, Yunnan, China; 3 Department of Hematology, Affiliated Hospital of Guizhou Medical University, Guizhou, China; 4 Department of Hematology, the General Hospital of Western Theater Command, Sichuan, China; 5 Department of Hematology, the Third Xiangya Hospital of Central South University, Hunan, China; 6 Department of Health Statistics, College of Military Preventive Medicine, Army Medical University, Chongqing, China; 7 Department of Epidemiology, College of Military Preventive Medicine, Army Medical University, Chongqing, China; 8 Department of Medicine, Keck School of Medicine, University of Southern California, Los Angeles; 9 Jinfeng Laboratory, Chongqing, China

## Abstract

**Importance:**

Chronic graft-vs-host disease (GVHD) limits the long-term benefit of haploidentical hematopoietic stem cell transplant (HSCT). This clinical trial evaluated repeated infusions of umbilical cord mesenchymal stem cells (MSCs) during the early stage (45 days and 100 days) after haplo-HSCT to prevent chronic GVHD.

**Objective:**

To determine whether repeated infusions of MSCs during the early stage after haplo-HSCT decreases the incidence of severe chronic GVHD.

**Design, Setting, and Participants:**

This open-label, multicenter, parallel randomized clinical trial was conducted from April 2016 to January 2022. Eligibility criteria included a diagnosis of acute leukemia and having a haploidentical, suitable related donor for HSCT. The median (range) follow-up time was 39.0 (1.5-67.0) months.

**Interventions:**

The enrolled patients with a haploidentical relative for HSCT received the modified busulfan/cyclophosphamide + antithymocyte globulin modified regimen and standard GVHD prophylaxis. Patients were randomly chosen to receive MSCs (the MSC group) (1 × 10^6^ cells/kg, every 2 weeks, starting from 45 days after transplant, 4 times total) or regular prophylaxis (control group).

**Main Outcome and Measure:**

The cumulative incidence of severe chronic GVHD.

**Results:**

Of 158 patients, 58 (36.7%) were female individuals; the median (range) age for the MSC and control groups was 28 (18-60) years and 28 (18-56) years, respectively. A total of 158 patients were screened, and 148 patients were randomly assigned to the MSC group (n = 74) or control group (n = 74) 1 day before MSCs infusion. The estimated 2-year cumulative incidence of severe chronic GVHD was 5.4% (95% CI, 1.8%-14.0%) in the MSC group and 17.4% (95% CI, 10.1%-28.5%) in the control group (hazard ratio [HR], 0.29; 95% CI, 0.10-0.88; *P* = .03). There was no difference between the MSC and control groups in the cumulative incidence of leukemia relapse (HR, 1.17; 95% CI, 0.55-2.47; *P* = .68). The cumulative incidence of stage II to IV acute GVHD in the MSC group was significantly lower than that in the control group (HR, 0.25; 95% CI, 0.09-0.67; *P* = .01). The MSC group had better GVHD-free and relapse-free survival rates than the control group (HR, 0.62; 95% CI, 0.39-0.98; *P* = .04).

**Conclusions and Relevance:**

The results of this randomized clinical trial show that early repeated infusions of MSCs decreased the incidence and severity of chronic GVHD, and the incidence and severity of acute GVHD manifested as a better GVHD-free and relapse-free survival rate for patients after haplo-HSCT.

**Trial Registration:**

Chinese Clinical Trial Registry: ChiCTR-IIR-16007806

## Key Points

**Question:**

Does infusion of mesenchymal stem cells during the early stage after haploidentical hematopoietic stem cell transplant help prevent chronic graft-vs-host disease?

**Findings:**

In this randomized clinical trial of 158 patients with acute leukemia, 4 rounds of prophylactic infusion of umbilical cord mesenchymal stem cells during 45 days to 81 days after haploidentical hematopoietic stem cell transplant significantly decreased the incidence and severity of chronic graft-vs-host disease as well as acute graft-vs-host disease. No effect on leukemia relapse or treatment-related adverse events was observed after repeated infusion of mesenchymal stem cells.

**Meaning:**

The results of this randomized clinical trial show that repeated mesenchymal stem cells infusion during the early stage after haploidentical hematopoietic stem cell transplant demonstrated clinically meaningful decline in severe chronic graft-vs-host disease occurrence and improvement in graft-vs-host disease-free and relapse-free survival without increasing the incidence of adverse events; however, further research on the MSCs infusion for haploidentical hematopoietic stem cell transplant complications management should be explored.

## Teaser

This randomized clinical trial examines whether repeated infusions of mesenchymal stem cells during the early stage after haploidentical hematopoietic stem cell transplant decreases the incidence of severe chronic graft-vs-host disease.

## Introduction

Chronic graft-vs-host disease (cGVHD) is the most common complication after allogeneic hematopoietic stem cell transplant (allo-HSCT) and substantially worsens quality of life.^1^ Based on the posttransplant cyclophosphamide (PTCy)–based and antithymocyte globulin (ATG)–based prophylactic regimens, the development of haploidentical allo-HSCT (haplo-HSCT) has been lifesaving for patients without human leukocyte antigen (HLA)–identical donors.^2^ However, HLA mismatch increases the risk of graft-vs-host disease (GVHD), as the incidence of acute GVHD (aGVHD) is 40% to 50% and the incidence of cGVHD is 50% to 60%. Better graft-vs-leukemia (GVL) effects have been shown in vivo and in samples from clinical trials.^3,4^ However, for patients after haplo-HSCT, the reported relapse rate is similar, and the overall survival (OS) rate is lower than that after HLA-identical HSCT due to the enhanced immune suppression needed to manage GVHD.^3,5,6^ For cGVHD, regardless of the therapy performed once cGVHD occurs, there is a mortality rate of more than 30% during follow-up.^7^ Since the therapeutic efficacy of mesenchymal stromal cells (MSCs) has been suggested in several clinical trials,^8,9^ MSCs could be a potential material to manage GVHD and preserve the GVL effect, which manifests as an improvement in the GVHD-free and relapse-free survival (GRFS) rate of transplant recipients.^10^ To efficiently prevent cGVHD, our team explored the cGVHD prophylactic efficacy of repeated infusions of MSCs starting 100 days after haplo-HSCT, which significantly decreased the incidence and severity of cGVHD in a randomized clinical trial (27.4% vs 49.0%; *P* = .02) that excluded almost all patients who developed a GVHD.^10^ According to the moderately good results of a meta-analysis of MSCs in GVHD, the timing of MSC infusion is very important and promotes different preventive effects.^11^ Thus, all patients with a risk of cGVHD were included in this study, which aimed to explore a novel infusion time in the early stage after transplant (45 days to 81 days) to include all patients who would potentially develop cGVHD.^12^

## Methods

### Trial Design

This trial was an open-label, multicenter, parallel randomized clinical trial that evaluated the effectiveness of MSC infusions for cGVHD prophylaxis after haplo-HSCT (Supplement 1). Patients were recruited between April 1, 2016, and January 1, 2020, after receiving approval from the ethics committees of 5 university hospitals in the Chinese Midwest. Written informed consent was provided by participants’ legal guardians and next of kin.

### Participants

Patients were recruited from the transplant centers of Xinqiao Hospital, Army Medical University, 920th Hospital of Joint Logistics Support Force, Affiliated Hospital of Guizhou Medical University, the General Hospital of Western Theater Command, and the Third Xiangya Hospital of Central South University. The physicians at the outpatient clinic who enrolled patients did not participate in the randomization and treatment. Eligible patients met the following criteria: (1) age between 18 and 60 years; (2) diagnosis of acute leukemia and failing in finding a HLA-matched related or unrelated donor and having an HLA-haploidentical suitable donor for HSCT; (3) a Karnofsky Performance Scale score of more than 60^13^ and a life expectancy of longer than 3 months; and (4) the absence of uncontrolled infections and of severe liver, kidney, lung, and heart diseases.

### Intervention

The enrolled patients with an HLA-haploidentical relative for HSCT received the mAy + ATG modified regimen.^14^ The donor selection and hematopoietic stem cell mobilization and collection was based on consensus from The Chinese Society of Hematology on indications, conditioning regimens, and donor selection for allogeneic hematopoietic stem cell transplant.^15^ The standard GVHD prophylaxis comprised mycophenolate mofetil, cyclosporine A, and methotrexate. The human umbilical cord (UC) was collected from healthy and full-term cesarean deliveries and processed within 24 hours after receiving signed informed consent by the third-party birth parent. Then, UC-MSCs were uniformly prepared from human UCs at Chongqing iCELL Biotechnology Co Ltd. Information on the production procedure and quality control of MSCs is described in the eAppendix of Supplement 2. The included patients, in a 1:1 ratio, were randomly chosen to receive MSCs (MSC group) (1 × 10^6^ cells/kg, every 2 weeks, starting from 45 days after transplant [4 times total]) or regular prophylaxis (control group).

### Outcomes

The primary outcome was the incidence of severe cGVHD. During follow-up, the overall incidence of cGVHD, incidence and severity of aGVHD, GRFS (survival without stage III to IV aGVHD, cGVHD that required systematic treatment, leukemia relapse, or death^16^), leukemia relapse, incidence and severity of an adverse event (AE) from 45 days to 100 days after haplo-HSCT, and OS were recorded as secondary outcomes. The outpatient clinic physicians who diagnosed and graded GVHD and adverse effects were unaware of the treatment allocation. We used the 2014 National Institutes of Health consensus criteria for organ scoring and the global assessment of chronic GVHD.^17^ We used Common Terminology Criteria for Adverse Events, version 4.0 (National Cancer Institute), to grade the AEs. In analyses of OS, death was counted as death of any reason after transplant.

### Randomization

A permuted block randomization method was used in this study. At each research center, consenting eligible participants were randomly assigned in a 1:1 ratio to the MSC group and control group using a computer-generated permuted block randomization schedule by statisticians who were not involved in the recruitment, treatment, and therapeutic effect evaluation (randomization in Supplement 2).

### Sample Size

Sample size estimates were based on the assumption of a log-rank test for the between-group comparison of the primary end point, which was the incidence of severe cGVHD. In the preliminary experiment, the cumulative incidence of severe cGVHD in the experimental and control group was 5.7% and 15%, respectively. We assumed 2 years for enrollment and 5 years for follow-up. A sample size of 142 patients was calculated with a 2-sided type 2 error of .05 and statistical power of 90% for the cumulative incidence of cGVHD. Considering the expected rate of loss to follow-up (10%), we decided to include 156 patients, with 78 patients in each arm. The sample size calculation was performed with PASS, version 15 (NCSS).^11^

### Statistical Analysis

The Mann-Whitney *U *tests, χ^2^ test, and Fisher exact test were used to compare the AEs between the MSC group and the control group. The competing risk model (Fine and Gray model) was used to estimate 2-year cumulative incidences and hazard ratios (HRs) with their 95% CIs for severe cGVHD and cGVHD (competed by death and relapse) and leukemia relapse (competed by nonrelapse death).^18^ The cumulative incidence of grade II to IV aGVHD and grade III to IV aGVHD, GRFS rate, and OS rate were estimated using Kaplan-Meier analysis and were expressed as percentages with 95% CIs. The Kaplan-Meier method, log-rank test, and Cox proportional hazard models were used to compare the grade II to IV aGVHD and III to IV aGVHD, GRFS, and OS curves between the 2 groups. All reported *P* values are 2-sided. Statistical analyses were performed using Stata, version 17 (StataCorp) and R (version 4.4.2; R Foundation for Statistical Computing).

## Results

### Patient Population and Disposition

**Figure 1.**
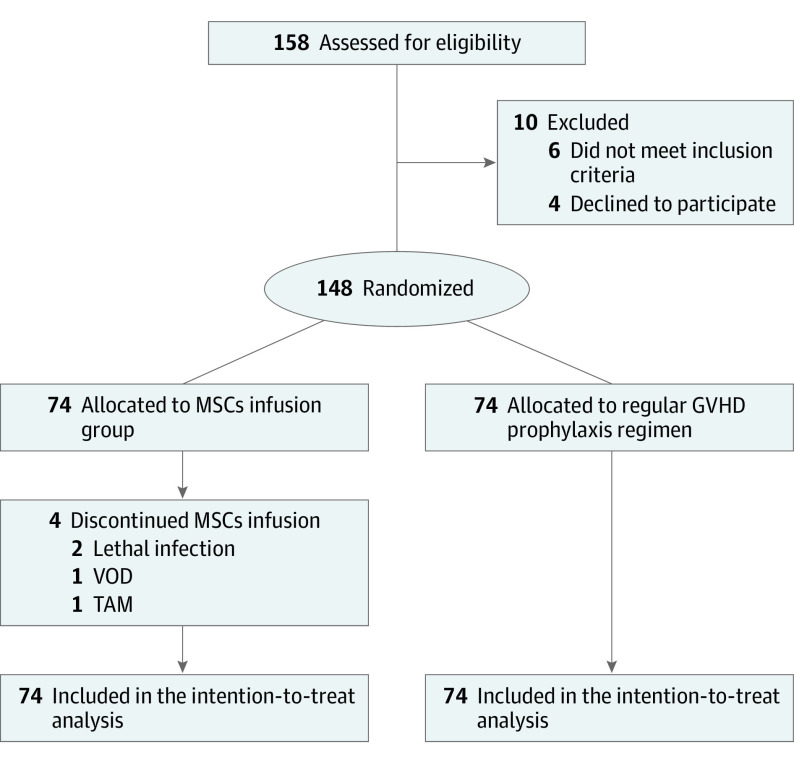
CONSORT Diagram of Study Participants GVHD indicates graft-vs-host disease; MSCs, mesenchymal stem cells; TAM, transplant-associated microangiopathy; VOD, veno-occlusive disease.

Between April 2016 and January 2022, 158 patients were screened at 5 transplant centers. Ten patients (6.3%) did not meet the inclusion criteria: 4 patients refused to participate in the study, 4 patients did not agree to sign the consent form, and leukemia relapse occurred in 2 patients before randomization. All participants received haploidentical HSCT with the haploidentical hematopoietic stem cells donated from a directive family (parent, child, or sibling). A total of 148 patients were randomly assigned to the MSC group (n = 74) or control group (n = 74) (1 day before the first MSC infusion). The median follow-up time was 39 (range, 1.5-67) months. The follow-up period ended in January 2022. Details of the study population and controls are reported in Figure 1. The most common reason for treatment discontinuation was leukemia relapse. In the MSC group, the prophylactic regimen was MSCs infusion regimen of 1 × 10^6^ cells/kg, every 2 weeks, starting 45 days after transplant for a total of 4 infusions for each patient, whereas the control group received a regular prophylactic regimen. Four patients did not complete the infusions because of lethal infection, venoocclusive disease, or transplant-associated microangiopathy at days 48, 49, 62, and 77 after haplo-HSCT, respectively. The baseline characteristics of evaluated patients are shown in the Table.

**Table.** Baseline Characteristics of Evaluated Patients

Characteristic	No. (%)	
	MSCs group (n = 74)	Control group (n = 74)
Age, median (range), y	28 (18-60)	28 (18-56)
Female	29 (39.2)	29 (39.2)
Male	45 (60.8)	45 (60.8)
HLA compatibility (A, B, and DR)		
1-Locus–mismatched	14 (18.9)	17 (23.0)
2-Locus–mismatched	28 (37.8)	22 (29.7)
3-Locus–mismatched	32 (43.3)	35 (47.3)
Disease		
AML	47 (63.5)	42 (56.8)
ALL	25 (33.8)	27 (36.5)
MAL	2 (2.7)	5 (6.7)
Donor-recipient sex match		
Female-female	3 (4.0)	4 (5.4)
Female-male	17 (23.0)	10 (13.5)
Male-male	29 (39.2)	37 (50.0)
Male-female	25 (33.8)	23 (31.1)
Donor-recipient relationship		
Parent to child	36 (48.7)	33 (44.6)
Child to parent	12 (16.2)	13 (17.6)
Siblings (haploidentical)	26 (35.1)	28 (37.8)

Abbreviations: ALL, acute lymphoblastic leukemia; AML, acute myeloid leukemia; HLA, human leukocyte antigen; MAL, mixed acute leukemia; MSCs, mesenchymal stem cells.

### cGVHD and Leukemia Relapse

**Figure 2.**
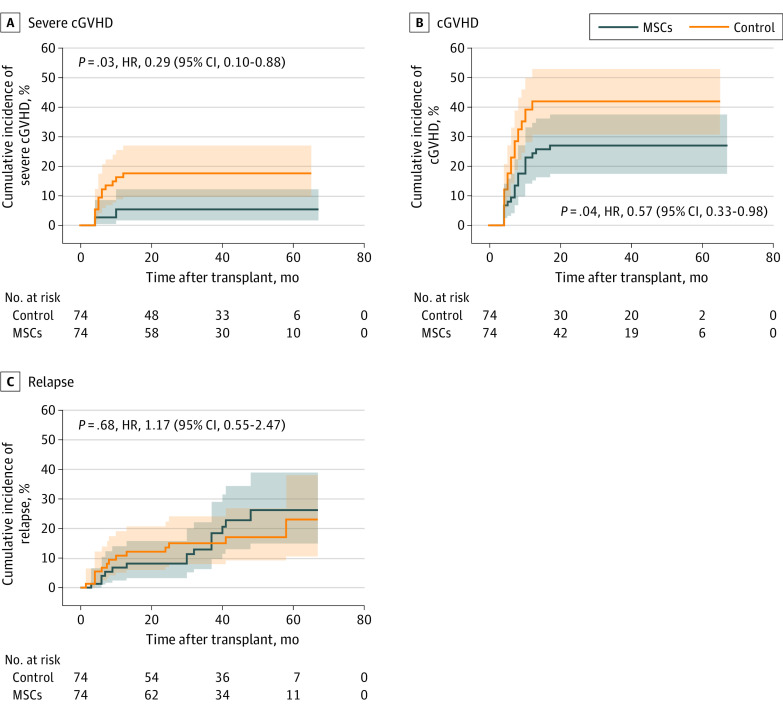
Cumulative Incidence of Severe Chronic Graft-vs-Host Disease (cGVHD), cGVHD, and Relapse HR indicates hazard ratio; MSCs, mesenchymal stem cells.

The estimated 2-year cumulative incidence of severe cGVHD was 5.4% (95% CI, 1.8%-14.0%) in the MSC infusion group and 17.4% (95% CI, 10.1%-28.5%) in the control group for an HR of 0.29 (95% CI, 0.10-0.88; *P* = .03) (Figure 2A). The estimated 2-year cumulative incidence of cGVHD was 15.8% (95% CI, 8.6%-26.6%) in the MSC infusion group and 26.2% (95% CI, 16.8%-37.9%) in the control group for an HR of 0.57 (95% CI, 0.33-0.98; *P* = .04) (Figure 2B). Moreover, in the lesion site analysis, 5 patients (6.8%; 95% CI, 2.9%-14.9%) developed liver cGVHD in the MSC group, while 15 (20.2%; 95% CI, 12.7%-30.8%) developed liver cGVHD in the control group (*P* = .01). Eleven patients (14.9%; 95% CI, 8.5%-24.7%) developed skin cGVHD in the MSC group, while 21 (28.4%; 95% CI, 19.4%-39.5%) developed skin cGVHD in the control group (*P* = .02). There were no significant differences in lung, joint, or fascia cGVHD between the MSC group and the control group. There was no significant difference between the MSC and control groups in the cumulative incidence of leukemia relapse (HR, 1.17; 95% CI, 0.55-2.47; *P* = .68) (Figure 2C). The incidence of severe cGVHD was not correlated with the mismatched HLA loci (HR, 1.23; 95% CI, 0.65-2.31; *P* = .52).

### aGVHD

**Figure 3.**
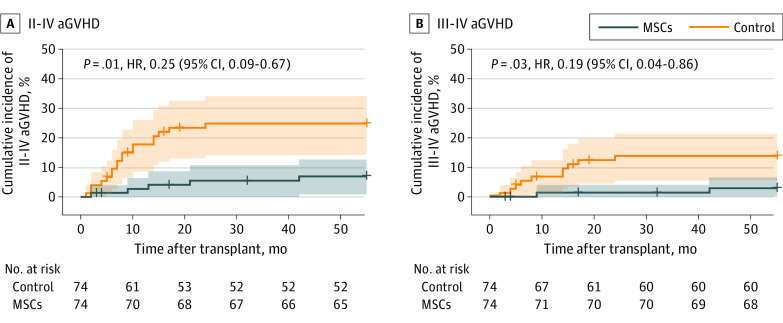
Cumulative Incidence of Grade II to IV Acute Graft-vs-Host Disease (aGVHD) and Grade III to IV aGVHD HR indicates hazard ratio; MSCs, mesenchymal stem cells.

A total of 11 patients (14.9%; 95% CI, 8.5%-24.7%) developed II-IV aGVHD in the MSC group, while 24 patients (32.4%; 95% CI, 22.9%-43.7%) developed II-IV aGVHD in the control group (*P* = .01). The incidence of grade III to IV aGVHD in the patients in the MSC group was significantly lower at 2.7% (95% CI, 0.5%-9.3%) compared with 13.5% (95% CI, 7.5%-23.1%; *P* = .01) in the control group. In grade II to IV aGVHD and III to IV aGVHD, the cumulative incidence curves of the MSC group were lower than those of the control group (HR, 0.25 [95% CI, 0.09-0.67; *P* = .01; Figure 3A] and 0.19 [95% CI, 0.04-0.86; *P* = .03; Figure 3B], respectively).

### GRFS and OS

**Figure 4.**
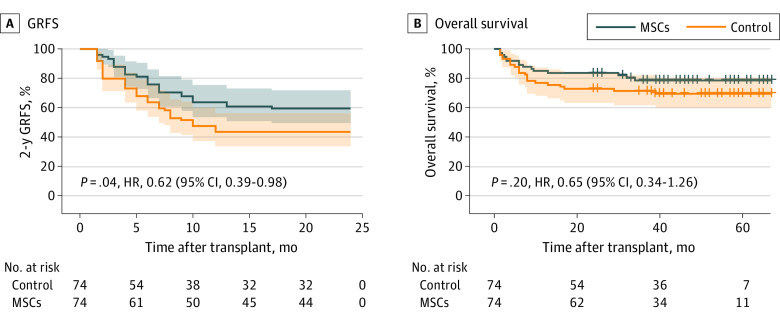
Two-Year Graft-vs-Host Disease–Free and Relapse-Free Survival (GRFS) and Overall Survival in the Mesenchymal Stem Cell (MSC) and Control Groups HR indicates hazard ratio.

The estimated 2-year GRFS was 59.5% (95% CI, 48.1%-69.9%) in the MSC infusion group and 26.2% (95% CI, 17.6%-37.2%) in the control group for an HR of 0.62 (95% CI, 0.39-0.98; *P* = .04) (Figure 4A).There was a potential improvement in the OS curve for the patients in the MSC group, although there was no significant difference between the groups (HR, 0.65; 95% CI, 0.34-1.26; *P* = .20) (Figure 4B). Furthermore, there were no cGVHD-related deaths in the MSC group, whereas 5 patients died of cGVHD-related complications in the control group.

### AE Analysis

All AEs that occurred between 45 and 100 days were recorded (eTables 1 and 2 in Supplement 2). The overall incidence of all grades of AEs was 82.4% (95% CI, 72.2%-89.4%) in the MSC group, while the incidence was 87.8% (95% CI, 78.5%-93.5%) in the control group (*P* = .36). The incidence of severe AEs was 44.6% (95% CI, 33.8%-55.9%) in the MSC group and 59.5% (95% CI, 48.1%-69.9%; *P* = .07) in the control group. There were no AEs observed that were related to the MSC infusion. In the kidney and genitourinary systems, there was a significant improvement in severe hemorrhagic cystitis; only 9 patients (12.2%; 95% CI, 6.5%-21.5%) developed grade III to IV hemorrhagic cystitis in the MSC group, while severe hemorrhagic cystitis occurred in 20 patients (27%; 95% CI, 18.2%-38.1%) in the control group (*P* = .02). As for infection, there were no significant differences in cytomegalovirus (CMV) viremia (37.8% vs 36.5%; *P* = .85) or Epstein–Barr virus (EBV) viremia (43.2% vs 41.9%; *P* = .87).

## Discussion

Haplo-HSCT has rapidly become more common in recent years, surpassing matched-sibling–donor HSCT, and has become the second most common source of allo-HSCT worldwide.^14^ With the development of PTCy-based and ATG-based prophylactic regimens, the incidence of cGVHD is approximately 50%, and the incidence of nonrelapse mortality (NRM) is approximately 30%, with cGVHD being the most common reason for NRM (37.8%).^19^ The current GVHD prevention methods, namely PTCy-based and ATG-based regimens, are mainly focused on combinations of immunosuppressors that are administered concurrently with hematopoietic stem cell infusion, which mainly have efficacy in aGVHD prophylaxis. A trial of low-dose PTCy with ATG/granulocyte colony-stimulating factor for patients undergoing haplo-HSCT decreased the cumulative incidence of grade III to IV aGVHD in the ATG-PTCy cohort to a rate significantly lower than that in the ATG group (5% vs 18%; *P* = .003) and increased the GRFS rate for patients who received low-dose PTCy.^20^ For cGVHD prophylaxis, the improved dose and duration of immunosuppressants increased the risk of organ dysfunction, infection, and recurrence.^21^ MSC therapy is a well-tolerated therapy for aGVHD and cGVHD. A recent retrospective clinical trial evaluated the prophylactic efficacy of the coinfusion of MSCs with HSCT in grade III to IV aGVHD (9.1% vs 22.8%; *P* = .03) and severe cGVHD (10.3% vs 22.2%; *P* = .04). However, there was no significant difference in the overall cases of aGVHD and cGVHD, and there was a trend of an increasing 3-year leukemia relapse rate (37.9% vs 26.7%; *P* = .19).^22^ To further improve the preventive effect of MSCs, we infused UC-MSCs earlier during the early stage after haplo-HSCT. There were significant reductions in the number of overall and severe cases of cGVHD, with a similar incidence in the leukemia relapse rate, with this protocol compared with the control protocol. Compared with other immune-suppressing GVHD prophylactic regimens, this regimen yielded a similar incidence of aGVHD and significantly improved the incidence of cGVHD. The present randomized clinical trial suggests the prophylactic efficacy of MSCs against cGVHD as well as aGVHD, which may enhance cGVHD prevention in the haplo-HSCT system.

Similar to most trials of GVHD prophylactic regimens, a nonsignificant improvement in OS was observed that could be related to the improvement in cGVHD treatment and smaller sample size. To evaluate the quality of life associated with GVHD, survival, and cancer management, GRFS was evaluated to measure the survival and quality of life of patients after allo-HSCT.^16^ In our study of infusion of MSCs for the prophylaxis of GVHD after haplo-HSCT, the significant reduction in the incidence and severity of aGVHD and cGVHD without influencing leukemia relapse resulted in a significant improvement in GRFS among the patients who received an MSC infusion. Current T cell–depleted haplo-HSCT with naive cell-enriched T cells has a 1-year GRFS rate of 56.5% based on the PTCy-based GVHD prevention regimen.^23^ The current study improves the GRFS rate for haplo-HSCT recipients based on ATG-based regimens.

The addition of traditional immunosuppressive drugs makes it difficult to avoid infection, especially viral infection.^24^ In our trial, there were no significant effects of infusion on CMV or EBV reactivation, both of which had a reactivation incidence of approximately 40% for both viral infections. The increased incidence of EBV (approximately 90%) in T cell–depleted haploidentical HSCT could lead to fatal lymphoproliferative disorders.^23^ In a trial of low-dose PTCy with ATG/granulocyte colony-stimulating factor, the CMV reactivation incidence was 73%, which requires an extra preemptive-like CMV–cytotoxic T lymphocyte strategy to prevent CMV disease.^20^ In this trial, the data indicated that infusion of MSCs did not increase the overall incidence of AEs and severe AEs. Furthermore, MSC infusion is beneficial in hemorrhagic cystitis management (12.2% vs 27.0%; *P* = .02), which could be explained by the tissue-repairing efficacy of MSCs. In general, MSC infusion is well tolerated and easily managed in clinical application and requires no other prophylactic or preemptive intervention for treatment-related complications.

Overall, repeated infusion of MSCs starting 45 days after haplo-HSCT is an appropriate strategy for cGVHD as well as aGVHD prophylaxis, and it successfully improves the GRFS of patients who have undergone haplo-HSCT. The possibility of administering MSCs right after transplant and the timing of later infusions should be explored to improve GVHD prophylactic efficacy and explore the efficacy of MSCs in HSC engraftment and tissue protection. Due to the positive characteristics and safety of MSCs, MSC infusion could be designed in combination with other GVHD prophylactic regimens, such as low-dose PTCy and T cell–depleted haploidentical HSCT, to further decrease the incidence of GVHD after haplo-HSCT and improve the life expectancy and quality of life of the recipients.

### Limitations

There was not a statistically significant difference between the OS of the 2 groups. We believe that this was because, first, the most common cause of death in both groups was leukemia relapse (10 patients per group), which was unrelated to MSCs infusion. Second, because the incidence of severe cGVHD is much higher than the incidence of cGVHD-related death, within the assumption of high inspection efficiency, the difference could be tested for severe cGVHD but not for OS. The limited trial scale is one of the main reasons why we saw no significant improvement in OS, which can be overcome with larger trials.

## Conclusions

In this phase 2 randomized clinical trial, 4 infusions of MSCs 45 days after haplo-HSCT reduced the incidence of severe cGVHD and improved GRFS. However, the improvement in OS requires a larger sample size for further verification.

## Notes

Supplement 1. Trial protocol

Supplement 2. eTable 1. Adverse events eTable 2. Serious adverse events eAppendix. Manufacture details of MSC products

Supplement 3. Data sharing statement
