# Supplementary material for: Mesenchymal Stem Cells for Prophylaxis of Chronic Graft-vs-Host Disease After Haploidentical Hematopoietic Stem Cell Transplant: An Open-Label Randomized Clinical Trial
Source: JAMA Oncol. 2023 Dec 28;10(2):220–6. doi: 10.1001/jamaoncol.2023.5757 (PMC10870190; doi:10.1001/jamaoncol.2023.5757)
Supplement: Supplement 1. — Trial protocol [file jamaoncol-e235757-s001.pdf]

Umbilical cord-derived mesenchymal cells prevent haploid-identical hematopoietic stem cell transplantation Graft-versus-host disease clinical research protocol

——Mesenchymal stem cells for prophylaxis of chronic graft-versus-host disease during the early stage after haploidentical hematopoietic stem cell transplantation

An Open-label, Multicenter, Randomized Controlled Clinical Trial

**Summary**	
Study name	Mesenchymal stem cells for prophylaxis of chronic graft-versus-host disease during the early stage after haploidentical hematopoietic stem cell transplantation<br>An Open-label, Multicenter, Randomized Controlled Clinical Trial
Clinical stage	Phase II clinical study
Research design	Multicenter, randomized, controlled
Research center	Medical Center of Hematology, Xinqiao Hospital, Army Medical University<br>920th Hospital of Joint Logistics Support Force<br>The Affiliated Hospital of Guizhou Medical University<br>The General Hospital of Western Theater Command<br>The Third Xiangya Hospital of Central South University
Test purposes	To explore the efficacy of umbilical cord-derived mesenchymal cells in preventing graft-versus-host disease (GVHD) after haploid-hematopoietic stem cell transplantation
Number of study cases	158 cases
Inclusion criteria	(1) Patients diagnosed with acute leukemia who underwent haploid hematopoietic stem cell transplantation;<br>(2) Gender is not limited, age 18-60 years old;<br>(3) KPS score > 60 points, estimated survival time > 3 months;<br>(4) Patients without serious systemic organ dysfunction (a. creatinine <1.5mg/dl; b. albumin ≥ 2; c. cardiac ejection index> 55%; d. hemoglobin> 9g/dL, bilirubin element <2.0mg/dl);<br>(5) The patient has no other contraindications for hematopoietic stem cell transplantation;<br>(6) Voluntary test, informed consent.
Exclusion criteria	(1) Severe cardiac, renal or hepatic insufficiency;<br>(2) Combined with other malignant tumors who need treatment;<br>(3) There are clinical symptoms of cerebral dysfunction or severe psychiatric disease, unable to understand or comply with the research protocol;<br>(4) Patients who cannot guarantee the completion of the necessary treatment plan and follow-up observation;<br>(5) Patients with severe acute allergic reactions;<br>(6) Clinically uncontrolled active infection;<br>(7) Patients who are participating in other clinical trials;<br>(8) The investigator believes that other reasons are not suitable for clinical trials.
Method of administration	1. On the basis of routine GVHD prevention in the experimental group, UC-MSCs were reinfused 45 days after transplantation at a dose of 1 × 10^6/kg per infusion; once every two weeks, a total of 4 times<br>2. Control group: routine GVHD prevention (Beijing program*) (MMF) 0.5g q12h -7d~90d, cyclosporine (CsA) 1.25mg/kg,; -1 day increment to 2.5mg/kg ; After the patient can tolerate orally, switch to 5 mg/kg orally, and the concentration is maintained at 200-300ng/ml; Slowly reduce the dose 180 days after transplantation; Methotrexate (MTX) 15mg/m2 d1, 10mg/m2 d3,6 ,11)
Observation period	(1) Real-time observation of the occurrence of acute and chronic GVHD after transplantation;<br>(2) Follow-up: relapse, death and HSCT related complication
Primary Endpoint	Severe chronic GVHD
Secondary Endpoint	Chronic GVHD, acute GVHD, Overall survival (OS), GVHD-free and Relapse-free survival (GRFS).

Safety evaluation	Incidence of adverse events (AE) and severe AE during 45 days to 100 days after haplo-HSCT
Study period	2016.4-2022.2

Investigator Statement

I have read this protocol and the research will be conducted in accordance with the moral, ethical and scientific principles stipulated in the Declaration of Helsinki and China GCP. I agree to carry out this clinical trial in accordance with the design and regulations of this protocol.

I will be responsible for making medical decisions related to clinical trials to ensure that subjects receive appropriate treatment in the event of adverse events during the trial. I know the procedures and requirements for proper reporting of serious adverse events, and I will record and report these events as required.

I guarantee that the data will be entered into medical records and case report forms in a true, accurate, complete, timely and lawful manner. I will accept the supervision or inspection of the monitor or the inspector dispatched by the sponsor and the inspection and inspection of the drug supervision and administration department to ensure the quality of the clinical trial.

I will provide a resume for submission to the ethics committee and possibly the regulatory authority before the study begins.

Principal Investigator (Signature) ____

Research investigation: Medical Center of Hematology, Xinqiao Hospital, Army Medical University

1. Research background

1.1 Background Introduction

Allogeneic hematopoietic stem cell transplantation (allo-HSCT) is an effective method for the treatment of acute leukemia. HLA-matched sibling donors are the most ideal choice, but the chance of matching is only 25%, rather than consanguinity. The probability of matching donors is only 1 in 50,000 to 100,000. HLA haploid hematopoietic stem cell transplantation (haplo-HSCT) effectively solves the problem of donor source. However, haploid transplantation that crosses the HLA barrier has a higher incidence of complications, of which graft versus host disease (GVHD) is the most important problem, and the incidence is significantly higher than that of HLA homozygous siblings Hematopoietic stem cell transplantation [1, 2] .

GVHD is a disease caused by immune cells (mainly T cells) contained in allografts that recognize the recipient's tissue antigens and launch an immune attack . It is the main complication of allo-HSCT and the main cause of death . According to the priority of onset, it is divided into acute GVHD (aGVHD: occurs within 100 days after transplantation) and chronic GVHD (cGVHD: occurs after 100 days) [3] . Acute GVHD has abrupt onset, rapid progression, and even life-threatening; chronic GVHD is prolonged and unhealed, seriously affecting the quality of life of patients. The treatment process of GVHD is long, the side effects of hormones or immunosuppressants are large, the incidence of severe infection is also significantly increased, and some patients have poor efficacy, so the prevention of GVHD is very important. At present, the prevention of GVHD is mainly to use immunosuppressive agents to remove lymphocytes in the transplant, but it also increases the recurrence of infection and leukemia, and the incidence of acute and chronic GVHD in haplo -HSCT patients is still as high as 50%; New methods for GVHD with high efficiency and low toxicity are of great significance.

Mesenchymal stromal cells (MSCs) are non-hematopoietic adult pluripotent stem cells first identified in the bone marrow stroma by Friedenstein in 1968 [4] . Studies have found that MSCs can maintain and increase the specific colony-forming units of CD34+ hematopoietic stem cells [5] , participate in regulating the growth of hematopoietic cells , and also have unique immunoregulatory properties. MSCs express moderate amounts of human leucocyte antigen (human leucocyte antigen , HLA) class I molecules , do not express HLA class II molecules , nor do they express FAS ligands and co-stimulatory molecules , such as B7-1 , B7-2 , CD40L, which can inhibit the Proliferative responses

of T cells in mixed lymphocyte cultures (MLCs) or mitogen stimulation. Animal experiments have found that MSCs from HLA-matched donors or " third parties " derived from expanded in vitro can prolong the survival time of allogeneic implantation into the skin after infusion into animals [6] , confirming that MSCs can play an immunosuppressive role in vivo.

The incidence of II-IV grade aGVHD after haploid hematopoietic stem cell transplantation is generally about 50% [7] , while the incidence of aGVHD in the treatment of refractory and recurrent hematological malignancies is only 24% after haploid hematopoietic stem cell transplantation combined with infusion of umbilical cord mesenchymal cells , suggesting that MSC combined infusion can prevent the occurrence of aGVHD [8] . In addition, our center proved for the first time internationally that the 2-year cumulative incidence of chronic GVHD after haplo -HSCT followed by sequential infusion of umbilical cord mesenchymal stromal cells (UC - MSCs) was 27.4%, which was significantly lower than 49.0% of the control group (P=0.021), showing that UC -MSCs can prevent the occurrence of cGVHD without increasing infection and tumor recurrence after transplantation [9] . The current studies on the prevention of GVHD by MSCs are all clinical studies on the prevention of aGVHD or cGVHD by MSCs alone. Based on the previous research, we plan to adopt the strategy of co-transplantation of UC-MSCs and HSCs and phased application of MSCs after transplantation to establish a safe and effective scheme that can prevent both aGVHD and cGVHD, and preliminarily elucidate its mechanism.

With the support of grants from the National Key R&D Program of China (2022YFA1103300, 2022YFA1103304), we designed a multi-center, prospective, randomized, controlled clinical study, and invited well-known domestic hematopoietic stem cell transplantation experts to conduct multiple demonstrations and formulate a research plan. For haplo-HSCT patients, the fourth-generation UC-MSCs were used without any genetic modification or gene editing. On the basis of routine GVHD prevention, the experimental group, on the basis of routine GVHD prevention, was infused with UC-MSCs 4 5 days after transplantation at a dose of 1×10^6/kg per infusion; once every two weeks, a total of 4 times . To explore the efficacy of UC-MSCs in preventing GVHD after haplo-HSCT , observe the recurrence rate after transplantation, transplantation-related complications and immune reconstitution, and preliminarily explore the relevant mechanisms. Explore new clinical and practical techniques for the prevention of GVHD after haplo-HSCT .

References

1. Baird K, Cooke K, Schultz KR , et al. Chronic Graft-Versus-Host Disease (GVHD) in Children. Pediatric Clinics of North America, 2010, 57(1): 297-322 .
2. Burke MJ, Trotz B, Luo X, et al. Allo-hematopoietic cell transplantation for Ph chromosome-positive ALL: impact of imatinib on relapse and survival. Bone Marrow Transplantation , 2009 , 43 (1) : 107-113 .
3. Fiuza-luces C , N uria garatachea N , S impson RJ , et al. Understanding graft-versus-host disease. Preliminary findings regarding the effects of exercise in affected patients . Exerc Immunol Rev , 2015; twenty one: 80-112.
4. Friedenstein AJ, Petrakova KV, Kurolesova AI, et al. Hetero typic transplants of bone marrow: analysis of precursor cells for osteogenic and hematopoietic tissues. Transplantation, 1968, 6 (2): 230 - 247.
5. Cheng L , Qasba P, Vanguri P, et al. Human mesenchymal stem cells support megacaryocyte and prop latelet formation from CD34+ hematopoietic progenitor cells. J Cell Physiol, 2000, 184 (1) : 58 - 59.
6. Krampera M , Glennie S, Dyson J, et al. Bonemarrow mesenchymal stem cells inhibit the response of naive and memory antigenspecific T cells to their cognate peptide. Blood, 2003, 101 (9): 3722-3729 .
7. Li Gao, Cheng Zhang, Lei Gao, et al. Favorable outcome of haploidentical hematopoietic stem cell transplantation in Philadelphia chromosome-positive acutelymphoblastic leukemia: a multicenterstudy in Southwest China. Journal of Hematology & Oncology , 2015 8:90 .
8. Wu Y , Wang Z , Cao Y , et al . Cotransplantation of haploidentical hematopoietic and umbilical cord mesenchymal stem cells with a myeloablative regimen for refractory/relapsed hematologic malignancy. Ann Hematol. 2013 , 92(12):1675-84.
9. Lei Gao, Yanqi Zhang, Baoyang Hu, et al. Phase II Multicenter, Randomized, Double-Blind Controlled Study of Efficacy and Safety of Umbilical Cord–DerivedMesenchymal Stromal Cells in the Prophylaxis of Chronic GraftVersus-Host Disease After HLA-Haploidentical Stem-Cell Transplantation. Journal of clinical oncology, 2016, 34:2843-2852.

2. Purpose of the clinical trial

To explore the efficacy and safety of umbilical cord-derived MSCs in preventing severe chronic graft-versus-host disease after haploid hematopoietic stem cell transplantation

3. Clinical trial design

3.1 Overall Design

A multicenter, randomized, controlled, open clinical trial was designed to explore the efficacy and safety of umbilical cord-derived MSCs in preventing graft-versus-host disease after haploid hematopoietic stem cell transplantation.

3.2 Criteria

3.2.1 Diagnostic criteria and grading criteria for acute graft-versus-host disease:

Acute graft-versus-host disease: a syndrome that occurs within 100 days of allogeneic hematopoietic stem cell transplantation and is characterized by rash, diarrhea, and cholestatic hepatitis. The diagnosis of acute graft-versus-host disease in the upper gastrointestinal tract requires endoscopic findings.

Clinical staging criteria for Seattle aGVHD

Staging	skin	liver bilirubin	gut
+	maculopapular rash <25% body surface area	2-3mg/dl	Diarrhea, 500-1000ml/day, or persistent nausea
++	maculopapular rash 25-50% body surface area	3-6mg/dl	Diarrhea, 1000-1500ml/day
+++	Systemic erythroderma	6-15mg/dl	Diarrhea, >1500ml/day
++++	peeling and bullae	>15mg/dl	abdominal pain and/or bowel obstruction

Seattle aGVHD

Grading	skin	liver	gut	loss of function
0 (none)	0	0	0	0
I (light)	+~++	0	0	0
II (moderate)	+~+++	+	+	+
III (severe)	++~+++	++~+++	++~+++	++
IV(Life Threatening)	+~++++	++~++++	++~++++	+++

3.2.2 Diagnostic criteria and grading criteria for chronic graft-versus-host disease

Chronic graft-versus-host disease: It occurs 100 days after transplantation, or is diagnosed as chronic GVHD with clinical manifestations of acute GVHD , without waiting for testing or other organ damage. The NIH working group believes that at least one chronic GVHD diagnostic feature and one unique manifestation can be diagnosed as chronic GVHD.

US NIH Chronic GVHD Consensus

Level	Degree of involvement
mild cGVHD	Damage to only 1 or 2 organs or sites (excluding lungs), no significant functional impact (level 1 damage)
Moderate cGVHD	At least 1 organ or site is damaged, but most of the function is still (level 2

	damage), or 3 or more organs or sites are damaged, but function is incomplete (level 1 damage), lung Partial damage level 1
Severe cGVHD	Most of the patient's function is impaired, and the degree of lung damage is grade 2 or above

3.3 Criteria for judging the efficacy of acute leukemia

Evaluation of the efficacy of acute leukemia: according to the 2011 edition of "Chinese Guidelines for Diagnosis and Treatment of Adult Acute Myeloid Leukemia (Non - Acute Promyelocytic Leukemia)" and the 2012 edition of "Expert Consensus on the Diagnosis and Treatment of Adult Acute Lymphoblastic Leukemia in China". Results included the following possibilities: complete remission (morphological complete remission, molecular complete remission, morphological complete remission without complete blood count recovery), relapse (molecular/genetic relapse, hematologic relapse) .

**Clinical efficacy**	**Evaluation indicators**
Morphological complete remission (CR)	Morphologically without leukemic status, free from transfusion, and without extramedullary leukemia. The absolute neutrophil count was $>1.0\times10^{9}$/L, and the platelets were $>100\times10^{9}$/L.
Cytogenetic complete remission (CRc)	Chromosomal return to normal karyotype after remission in patients with chromosomal abnormalities before treatment
Molecular complete remission (CRm)	Patients with specific genetic markers and immunophenotypic characteristics before treatment turned negative after treatment
Morphological complete remission without complete blood count recovery (CRi)	Meets clinical and myeloid criteria for CR but still has neutropenia ($<1.0\times10^{9}$/L) or thrombocytopenia ($<100\times10^{9}$/L)
Morphological recurrence	Recurrence of leukemia cells in the peripheral blood of CR patients, immature cells in the bone marrow ≥ 5% or new pathological hematopoiesis, and morphologically verifiable leukemia cells in the extramedullary
Molecular/genetic recurrence	Cytogenetic or molecular abnormalities in patients who have achieved complete remission at the cytogenetic or molecular level

3.4 Criteria for judging transplantation-related complications

Various toxic and side effects occurred in patients after haploid hematopoietic stem cell transplantation were observed. According to various adverse reactions that may occur after treatment, the adverse reactions were divided into 5 grades to evaluate the side effects of the treatment in this study.

**Grading criteria for adverse events in haploid hematopoietic stem cell transplantation (refer to CTCAE v4.0)**	
	**level**

	Level 1	level 2	Level 3	level 4	Level 5
**Blood disease adverse events**					
Anemia	Hemoglobin < lower limit of normal - 100g/L	Hemoglobin <100-80g/L	Hemoglobin <80-65g/L; blood transfusion required	Life-threatening consequences; urgent medical attention required	Death
Decreased lymphocyte count	<LLN-800/mm3; <LLN×0.8×10^9/L	<800-500/mm3; <0.8-0.5×10^9/L	<500- 200/mm3; <0.5-0.2×10^9/L	<200/mm3; <0.2×10^9/L	Death
Decreased neutrophil count	<Lower limit of normal value-1500/mm^3 ; <Lower limit of normal value-1.5×10^9/L	<1500- 1000/mm^3 ; <1.5-1.0× 10^9/L	<1000- 500/mm3; <1.0-0.5× 10^9/L	<500/mm3; <0.5×10^9/L	Death
Decreased platelet count	<Lower limit of normal value-75,000/mm^3; < Lower limit of normal value-75.0×10^9/L	<75,000-50,000/mm^3; <75.0-50.0 ×10^9/L	<50,000-25,000/mm^3; <50.0-25.0 ×10^9/L	<25,000/mm^3 ; <25.0×10^9/L	Death
Decreased white blood cell count	<Lower limit of normal value-3000/mm^3 ; <Lower limit of normal value-3.0×10^9/L	<3000- 2000/mm^3; <3.0-2.0×10^9/L	<2000-1000/mm^3 ; <2.0-1.0× 10^9/L	<1000/mm3; <1.0×10^9/L	Death
**Laboratory abnormalities**					
Elevated alanine aminotransferase (ALT)	>Upper Normal - 3.0x Upper Normal	Asymptomatic: >3.0-5.0 times the upper limit of normal; >3 times the upper limit of normal, accompanied by aggravation of the following symptoms: fatigue, nausea, vomiting, pain or tenderness in the upper right area, fever, rash, eosinophilia	>5.0-20.0 times the upper limit of normal; for more than 2 weeks, >5 times the upper limit of normal	>20.0 times the upper limit of normal	Death
Elevated aspartate aminotransferase (AST)	>Upper Normal - 3.0× Upper Normal	Asymptomatic: >3.0-5.0 times the upper limit of normal; >3 times the upper limit of normal with aggravation of the following symptoms: fatigue, nausea, vomiting, pain or tenderness in the upper right	>5.0-20.0 times the upper limit of normal; for more than 2 weeks, >5 times the upper limit of normal	>20.0 times the upper limit of normal	Death

		area, fever, rash, eosinophilia			
Increased blood bilirubin	>Upper Normal - 1.5x Upper Normal	>1.5-3.0 times the upper limit of normal	>3.0-10.0 times the upper limit of normal value	>10.0 times the upper limit of normal	Death
Increased creatine phosphokinase (CPK)	>Upper Normal - 2.5x Upper Normal	>2.5 times the upper limit of normal value - 5 times the upper limit of normal value	>5 times the upper limit of normal value - 10 times the upper limit of normal value	>10 times the upper limit of normal	Death
Hyperglycemia	Fasting glucose concentration > upper limit of normal - 160 mg/dL; fasting glucose concentration > upper limit of normal - 8.9 mmol/L	Fasting glucose concentration >160-250 mg/dL; fasting glucose concentration >8.9-13.9 mmol/L	>250-500 mg/dL; >13.9-27.8 mmol/L; hospitalization required	>500mg/dL; >27.8mmol/L; life-threatening	Death
Hypokalemia	<Lower limit of normal value -3.0 mmol/L	<lower limit of normal -3.0 mmol/L ; asymptomatic: treatment required	<3.0-2.5 mmol/L; hospitalization required	<2.5 mmol/L; life-threatening	Death
hyponatremia	<Lower limit of normal value -130mmol/L	--	<130-120mmol/L	<120mmol/L; life-threatening	Death
hypophosphatemia	< lower limit of normal - 2.5 mg/dL; < lower limit of normal - 0.8 mmol/L	<2.5-2.0 mg/dL; <0.8-0.6 mmol/L	<2.0-1.0 mg/dL; <0.6-0.3mmol/L	<1.0 mg/dL; <0.3 mmol/L ; life-threatening	Death
Decreased fibrinogen	<1.0-0.75 times lower limit of normal or 25% reduction from baseline	<0.75-0.5 times lower limit of normal or 25%-<50% reduction from baseline	<0.5-0.25 times lower limit of normal or 50-<75% reduction from baseline	<0.25 times lower limit of normal or 75% reduction from baseline, or absolute value <50 mg/dL	Death

4. Subject selection

4.1 Inclusion criteria

(1) Patients diagnosed with acute leukemia and undergoing haploid hematopoietic stem cell transplantation, regardless of gender, aged 18-60 years; KPS score > 60 points, expected survival > 3 months;

(2) Those who do not have serious damage to the function of important organs of the body;

(3) Signed the informed consent.

4.2 Exclusion criteria

(1) Severe cardiac, renal or hepatic insufficiency;

(2) Combined with other malignant tumors that need treatment ;

(3) There are clinical symptoms of brain dysfunction or severe psychiatric disease that cannot be understood or comply with the research protocol ;

(4) Those who cannot follow up as scheduled ;

(5) Patients who are not guaranteed to complete the required treatment plan and follow-up observation.

4.3 Rejection or shedding criteria:

During the treatment and follow-up period, disease changes or death unrelated to the experimental factors occurred, and the observation could not be continued.

4.4 Termination criteria:

(1) Severe adverse reactions intolerable;

(2) The subject voluntarily requests to withdraw;

(3) Relapsed before receiving the MSC infusion;

(4) The investigators considered that the subjects were not suitable to continue treatment for other reasons.

5. Treatment options

5.1 GVHD Prevention

(1) Experimental group: On the basis of conventional GVHD prevention, the experimental group was re-infused with UC-MSCs 45 days after transplantation at a dose of 1×10^6/kg; infusion every two weeks once, 4 times

(2) Control group: conventional GVHD prevention (Beijing program) (MMF) 0.5g q12h -7d~90d, cyclosporine (CsA) 1.25mg/kg,; -1 day increment to 2.5mg/ kg; after the patient can tolerate it orally, it is changed to 5 mg/kg orally, and the concentration is maintained at 200-300ng/ml; the dose is slowly reduced 180 days after transplantation; methotrexate (MTX) 15mg/m ^{2} d1, 10mg/m ^{2} d3,6,11)

5.2 Indicator observation

(1) The incidence and severe chronic GVHD

(2) The overall incidence of chronic GVHD The incidence and severity of acute GVHD, OS , Post-transplant patient GRFS (GVHD-free and Relapse Free Survival)

6. Research Process

6.1 Recruitment and Baseline Assessment

Subjects who meet the conditions of 4.1 of this research protocol will undergo a series of functional examinations as necessary research indicators for clinical research. The physicians at the outpatient clinic who recruited patients did not participate in the randomization and treatment. The main examination items include but are not limited to the following examination items:

(1) Physical examination (weight, height, body surface area)

(2) Vital signs check: temperature, pulse, heart function check

(3) Routine blood tests: hemoglobin, white blood cells, platelets, etc.

(4) Liver and kidney function tests

(5) bone marrow imaging, bone marrow biopsy

(6) Leukemia residual disease (MRD), related gene mutation detection

6.2 Collection of subjects' documents and materials

(1) Documents required to recruit subjects include:

(2) Complete the required materials for screening;

(3) Sign the informed consent;

6.3 Implementation of haploid hematopoietic stem cell transplantation

6.3.1 Preprocessing scheme Me-CCNU+Ara-C+Bu+Cy+ATG (Beijing protocol)

Me-CCNU:250mg/m ^{2} ×1d, Ara-C: 4g/m ^{2} ×2d, Bu: 0.8mg/kg q6h, ×2d, CTX: 1.8g/m ^{2} ×2d

6.3.2 Hematopoietic stem cell mobilization, collection and reinfusion

The donors were given subcutaneous injection of recombinant human granulocyte colony-stimulating factor (rhGCSF) at 10 u g/kg / d for 4 days, and peripheral blood stem cells (peripheral blood stem cells) were collected by a German Fresenius blood cell separator on d5 . blood stem cells , PBSC) . On day 6 , bone marrow collection was performed. After epidural anesthesia was performed, the donor was placed prone on the operating bed, and puncture was performed at different depths and multiple sites on the bilateral iliac ridges for collection; if it is estimated that more than 600ml of bone marrow fluid needs to be collected, the donor should be adjusted to the supine position, collected in bilateral anterior superior iliac spine puncture. If the donor and recipient ABO blood type is incompatible on the primary side, erythrocytes are removed by hydroxyethyl starch sedimentation; if the secondary side is incompatible, the bone marrow fluid needs to be centrifuged at 4°C at a low temperature, and then the plasma in the bone marrow fluid is removed with a plasma separator.

6.4 Prevention of GVHD after haploid hematopoietic stem cell transplantation

(1) Experimental group: On the basis of conventional GVHD prevention, UC-MSCs were infused 45 days after transplantation at a dose of 1×10^6/kg with the interval of 14 days for 4 times.

(2) Control group: Beijing program for routine GVHD prevention (MMF) 0.5g q12h -7d~90d, cyclosporine (CsA) 1.25mg/kg, continuous intravenous drip; -1 day increment to 2.5mg/kg; After the patient can tolerate oral administration, it is changed to oral 5 mg/kg, and the concentration is maintained at 200-300 ng/ml; the dose is slowly reduced 180 days after transplantation; methotrexate (MTX) 15 mg/m 2 d1, 10 mg/m 2 d3, 6,11)

6.5 Indicator observation

(1) The incidence of severe chronic GVHD

(2) The incidence of II-IV acute GVHD, the incidence of chronic GVHD, OS, Post-transplant patient GRFS (GVHD-free and relapse-free survival)

6.6 Therapeutic Monitoring Protocol

After hematopoietic reconstruction to 1 year after transplantation, clinicians were blinded to the treatment allocation will access:

(1) Routine blood tests, liver and kidney function tests, CMV and EBV and other viral indicators (at least monthly)

(2) Bone marrow imaging, bone marrow biopsy (monthly)

(3) MRD, related gene mutation detection (monthly)7. Safety and Adverse Events

Safety will be measured and assessed daily by documenting potential adverse effects of treatment. Patients will be tested for potential toxicity that may arise from the treatment through tests such as medical history, physical examination, blood tests, etc.

6.7 Recording of Adverse Events

Investigators can gather information on adverse events through special questions or, in certain circumstances, tests in their interactions with each patient. All information about adverse events needs to be entered into the database immediately and into the Adverse Events module of the Case Report Form (CRF). All apparently relevant signs, symptoms, and abnormal diagnoses need to be entered into the database and grouped under the same diagnosis.

All adverse events that occurred during the study period were required to be recorded. All adverse events needed to be followed until resolved, stabilized, or until confirmed that study treatment or

participation was not an issue. Serious adverse events that persisted at the end of the study had to be followed until final results were determined. Any serious adverse events that occur after the study and that may be related to study treatment or study participation need to be recorded and reported promptly. If any subject has the following conditions within 2 weeks of receiving cell reinfusion therapy , the study will be required to be re-reviewed, and the clinical trial at this stage will be suspended:

(1) Respiratory failure requires artificial respirator.

(2) Grade 4 (life-threatening) toxicity due to treatment regimen.

(3) Death.

7. Randomization

A permuted block randomization method was used in this study. At each research center, consenting eligible participants were randomly assigned in a 1:1 ratio to the MSC group and control group using a computer-generated permuted block randomization schedule by statisticians who were not involved in the recruitment, treatment and therapeutic effect evaluation.. The permuted block size was randomly generated from among the numbers 4 and 6.

With permuted block of size four there are six possible patterns: AABB, ABAB, ABBA, BAAB, BABA, and BBAA.

With permuted block of size six there are 20 possible patterns:

AAABBB	ABABAB	BAAABB	BABBAA
AABABB	ABABBA	BAABAB	BBAAAB
AABBAB	ABBAAB	BAABBA	BBAABA
AABBBA	ABBABA	BABAAB	BBABAA
ABAABB	ABBBAA	BABABA	BBBAAA

8. Sample Size

Sample-size estimates were based on the assumption of a log-rank test for the between-group comparison of the primary end point, the incidence of cGVHD. In the preliminary experiment, the cumulative incidence of severe cGVHD in the experimental and control group was 5.7% and 15%, respectively. We assumed 2 years for enrollment and 5 years for follow-up. A sample size of 142 total patients was calculated with a two-sided type II error of 0.05 and a statistical power of 90% for the

cumulative incidence of cGVHD. Considering the expected rate of loss to follow-up (10%), we decided to include 158 patients, with 79 patients in each arm. The sample size calculation was performed with PASS version 15 software (NCSS, Kaysville, UT).

9. Ethical requirements

This clinical trial will be conducted in accordance with the Declaration of Helsinki and Good Clinical Practice (GCP).

9.1 Ethics Committee

Before the start of the clinical trial, the clinical trial protocol needs to be reviewed and approved by the ethics committee and signed for approval before it can be implemented. During the clinical trial, any modification of the clinical trial protocol and informed consent form must be approved by the ethics committee before it can be implemented.

9.2 Informed Consent

This clinical trial will provide all subjects with an informed consent form describing the study and provide sufficient information to allow subjects to make an informed decision about whether or not to participate in the study. This informed consent form will be submitted to the ethics committee for review and review together with the clinical trial process document. After the ethics committee has reviewed the informed consent form, subjects must sign the approved version of the informed consent form before entering any clinical trial. The informed consent form must be signed by the subjects themselves or their legal representatives, and the research professionals designated by the investigator will collect the informed consent form.

10. Subject management

During the treatment period, the subjects will be followed up by a special investigator, and if they feel unwell, they can contact the investigator at any time to ensure the safety of the patients to the greatest extent.

The subject's treatment compliance includes the subject's consent to participate in the study, willingness to receive the prescribed drug treatment, and willingness to collect blood samples and bone marrow specimens to evaluate efficacy and safety. Subjects may be excluded from the study due to lack of good compliance with treatment or follow-up at the discretion of the sponsor and investigator.

11. Statistical Analysis Plan

The Mann–Whitney tests, the chi-squared test, and Fisher's exact test were used to compare the AE between the MSC group and the control group. The competing risk model (Fine and Gray model) was used to estimate 2-year cumulative incidences and hazard ratios (HRs), with their 95% CIs, for severe cGVHD and cGVHD (competed by death and relapse) and leukemia relapse (competed by nonrelapse death). The cumulative incidence of grade II-IV aGVHD and grade III-IV aGVHD, the GRFS rate and the OS rate were estimated using Kaplan–Meier analysis and are expressed as percentages with 95% CIs. The Kaplan–Meier method, the log-rank test, and Cox proportional hazard models was used to compare the grade II-IV aGVHD and III-IV aGVHD, GRFS and OS curves between the two groups. All reported *P* values are two-sided. Statistical analyses were performed using STATA 17 (StataCorp. 2021. Stata Statistical Software: Release 17. College Station, TX: StataCorp LLC)) and R (version 4.4.2, R Foundation for Statistical Computing, Vienna, Austria).
