# Supplementary material for: Mesenchymal Stem Cells for Prophylaxis of Chronic Graft-vs-Host Disease After Haploidentical Hematopoietic Stem Cell Transplant: An Open-Label Randomized Clinical Trial
Source: JAMA Oncol. 2023 Dec 28;10(2):220–6. doi: 10.1001/jamaoncol.2023.5757 (PMC10870190; doi:10.1001/jamaoncol.2023.5757)
Supplement: Supplement 2. — eTable 1. Adverse events eTable 2. Serious adverse events eAppendix. Manufacture details of MSC products [file jamaoncol-e235757-s002.pdf]

Supplemental Online Content

Huang R, Chen T, Wang S, et al. Mesenchymal stem cells for prophylaxis of chronic graft vs-host disease after haploidentical hematopoietic stem cell transplant: an open-label randomized clinical trial. *JAMA Oncol*. Published online December 28, 2023.
doi:10.1001/jamaoncol.2023.5757

eTable 1. Adverse events
eTable 2. Serious adverse events
eAppendix. Manufacture details of MSC products

This supplemental material has been provided by the authors to give readers additional information about their work.

© 2023 Huange R et al. *JAMA Oncology.*

Table 1. Adverse Events

**Event**	**MSCs group (n=74) Any grade**	**Control group (n=74) Any grade**	***P* value**
Platelets decreased	14 (18.9%)	18 (24.3%)	.42
Neutrophils decreased	10 (13.5%)	13 (17.6%)	.50
Gastrointestinal[1]	20 (27.0%)	24 (32.4%)	.47
Liver[1]	12 (16.2%)	17 (23.0%)	.30
Bacterial infections	15 (20.3%)	17 (23.0%)	.69
Fungal infections	10 (13.5%)	15 (20.3%)	.27
Viremia	44 (59.5%)	44 (59.5%)	>.99
Cardiac	2 (2.7%)	4 (5.4%)	.68
Kidney and Urinary system	16 (21.6%)	25 (33.8%)	.10

[1]Excluded patients with cGVHD

© 2023 Huange R et al. *JAMA Oncology.*

eTable 2. Serious Adverse Events

**Event**	**MSCs group (n=74)** **Any grade**	**Control group (n=74)** **Any grade**	***P* value**
Platelets decreased	4 (5.4%)	9 (12.2%)	.15
Neutrophils decreased	4 (5.4%)	5 (6.8%)	>.99
Gastrointestinal[1]	1 (1.4%)	2 (2.7%)	>.99
Liver[1]	4 (5.4%)	6 (8.1%)	.51
Bacterial infections	13 (17.6%)	13 (17.6%)	>.99
Fungal infections	8 (10.8%)	10 (13.5%)	.62
Viremia	13 (17.6%)	21 (28.4%)	.12
Cardiac	0 (0%)	1 (1.4%)	>.99
Kidney and Urinary system	9 (12.2%)	20 (27.0%)	.02

[1]Excluded patients with cGVHD

© 2023 Huange R et al. *JAMA Oncology.*

eAppendix.

Manufacture details of MSC products

Rinse fresh healthy umbilical cord with PBS, then remove the blood vessels and peel out the Waldorf glue tissue, cut the obtained tissue to 1 mm3 size, add the culture medium in 37°C, 5% CO_2 incubator, the culture medium contains 10% serum substitute. After 14 days of culture, digest the cell colonies with 0.25% trypsin for passaging. The approximate 5×10^{10} of P4 generation cells can be cultured per umbilical cord. The quality control is as below.

**Items**		**Standard**
Appearance		Colorless translucent cell suspension<br>No foreign matter or granular material
Cell Viability		⩾90%
Cell Phenotypic	CD73	⩾95%
	CD90	⩾95%
	CD105	⩾95%
	CD34	⩽2%
	CD45	⩽2%
	HLA-DR	⩽2%
Bacteria		Negative
Fungi		Negative
Mycoplasma		Negative
Endotoxin		0.5EU/ml

© 2023 Huange R et al. *JAMA Oncology.*
