# Supplementary material for: Mesenchymal Stem Cells for Prophylaxis of Chronic Graft-vs-Host Disease After Haploidentical Hematopoietic Stem Cell Transplant: An Open-Label Randomized Clinical Trial
Source: JAMA Oncol. 2023 Dec 28;10(2):220–6. doi: 10.1001/jamaoncol.2023.5757 (PMC10870190; doi:10.1001/jamaoncol.2023.5757)
Supplement: Supplement 3. — Data sharing statement [file jamaoncol-e235757-s003.pdf]

**Data Sharing Statement**

**Data**
**Data available:** Yes
**Data types:** Deidentified participant data
**How to access data:** Please email the corresponding author for data regarding this clinical trial. zhangxxi@sina.com.
**When available:** With publication

**Supporting Documents**
**Document types:** None

**Additional Information**
**Who can access the data:** Please email the corresponding author for data regarding this clinical trial. zhangxxi@sina.com.
**Types of analyses:** Researchers whose proposed use of the data has been approved.
**Mechanisms of data availability:** With a signed zhangxxi@sina.com. Please email the corresponding author for data regarding this clinical trial, data will be released in compliance with institutional policy and agreement.
